# Suicidality in Fibromyalgia: A Systematic Review of the Literature

**DOI:** 10.3389/fpsyt.2020.535368

**Published:** 2020-09-23

**Authors:** Daniella Levine, Danny Horesh

**Affiliations:** ^1^ Department of Psychology, New York University, New York, NY, United States; ^2^ Department of Psychiatry, Massachusetts General Hospital, Boston, MA, United States; ^3^ Department of Psychology, Bar-Ilan University, Ramat Gan, Israel; ^4^ Department of Psychiatry, New York University Langone Medical Center, New York, NY, United States

**Keywords:** fibromyalgia, suicidality, chronic pain, review, suicide, suicidal ideation, suicidal behavior

## Abstract

Fibromyalgia (FM), a poorly understood rheumatic condition, is characterized by chronic pain and psychiatric comorbidities, most notably depression and anxiety. Additional symptoms include sleep difficulties, fatigue, and various cognitive impairments. Furthermore, FM is surrounded by social stigma, due to the unclear nature and etiology of this condition. While there is widespread evidence for the emotional and psychological suffering of those with FM, the scope of suicidality, as well as the underlying factors that are associated with suicidal ideation and behavior among this population, are not well understood. The present review, which is the first of its kind, aims to summarize existing data on the prevalence of suicide-related outcomes among FM patients, highlight factors associated with suicidal ideation and behavior in FM, and identify gaps in the literature to better inform research and clinical care. Studies were extracted from the literature that measured suicidal ideation, attempted suicide, and/or completed suicide among FM patients. Results indicated that both suicidal ideation and suicidal behavior were prevalent among individuals suffering from FM. Psychiatric comorbidity, sleep difficulties, and inpatient hospitalization were associated with both suicidal ideation and suicidal behavior. Functional impairment was associated with suicidal ideation in FM. Factors associated with higher levels of suicidal behavior in FM included female gender, unemployment and lower income, medical comorbidity, and drug dependence. While an understanding of currently recognized risk factors is important for improving FM research and clinical care, some clear methodological and conceptual limitations of the reviewed studies were identified. Future work should focus on longitudinal studies, as well as on gaining a better biological and psychological understanding of the underpinnings of FM and suicidality.

## Introduction

Fibromyalgia (FM) is a chronic rheumatic condition that causes widespread pain throughout the body. Current research suggests that FM is centralized, meaning that the amplification of pain originates in the central nervous system (1). Originally defined in 1990 by the American College of Rheumatology (ACR), FM was diagnosed if a patient had chronic widespread pain in at least 11 out of 18 “tender points” (e.g. pain at the lateral pectorals, the upper quadrant of the buttocks, the knees), as well as endorsed additional symptoms such as sleep disturbance, fatigue, and morning stiffness (2). Widespread pain was specified to be axial pain, left or right-sided pain, or upper or lower segment pain (2). Diagnostic criteria were modified by the ACR in 2010 and 2011, first shifting the diagnostic focus away from tender points and towards a clearer and more specific variety of symptoms, then allowing for the possibility of self-reported diagnosis in a research setting, as well as adding a fibromyalgia severity score (3). According to these newly defined criteria, FM is diagnosed if a patient reports widespread pain in at least one of 19 musculoskeletal regions as assessed by a Widespread Pain Index score greater than or equal to 7 (4), as well as core symptoms of fatigue, waking unrefreshed, somatic symptoms, and cognitive disturbances as assessed by a Symptom Severity Scale greater than or equal to 5 (5). Patients can also satisfy 2010 diagnostic criteria if they have a Widespread Pain Index score between 3–6 and a Symptom Severity Scale score greater than or equal to 9 (6). Other symptoms include memory difficulties, headaches, irritable bowel movements, and mood disturbances (4). Symptoms must have a duration of at least three months (6). The criteria were then changed again in 2016, when widespread pain scores were modified, a generalized pain criterion was added, symptoms reported across patients and physicians were standardized, and wording of symptom duration was standardized. Most notably, whereas the previous diagnosis of fibromyalgia was conditional upon the lack of any other disorder that could potentially explain the chronic pain, in the newest diagnostic criteria it was ascertained that an individual could have FM while still suffering from other pain conditions (3).

Cognitive difficulties, while not commonly assessed at diagnosis, are also common in FM. These include “Fibro Fog,” or dyscognition, defined as cognitive dysfunction characterized by memory lapses, confusion, as well as impaired concentration, planning, and organization (7). Fibro Fog is experienced by 76.4–82.5% of patients with FM (8), yet dyscognition was only added to the ACR diagnostic criteria in 2010 (5).

Prevalence rates of FM have been found to vary between 0.2–4.7% (e.g., 9). Among women, prevalence rates range between 2.4–6.8% (9), with about a 9:1 female-to-male prevalence ratio (10), although one study found slightly less of a distinct gender difference (11). Interestingly, in one recent study conducted by Wolfe and colleagues (12) among a sample of 2,445 adults, no significant gender difference in FM prevalence rates was found. One explanation concerning this disparity could be the changing diagnostic criteria. The reliance of the original ACR criteria on tender points may have resulted in higher FM rates among women, as women were found to have more tender points than men (5). Since Wolfe and colleagues (12) utilized the modified ACR criteria, which rely on tender points to a lesser degree, the gender ratios may have become more proportionate.

Oftentimes, FM co-exists with other medical and mental disorders. Among medical disorders and diseases, it has been found that patients with FM are significantly more likely to suffer from diabetes, hypertension, hyperlipidemia, congestive heart failure, cerebrovascular disease, irritable bowel syndrome, headaches, and chronic liver disease when compared to patients not suffering from FM (13). Another study found that patients with FM exhibited high rates of migraines, irritable bowel syndrome, and chronic fatigue syndrome (14). In terms of psychiatric comorbidities, studies show that patients with FM are significantly more likely to suffer from major depression, anxiety, and sleep disorders when compared to patients not suffering from FM (13).

Major depression in particular has been found to be 20–60% more prevalent among patients with FM when compared to the general population (15). This increased prevalence may be attributed to several factors, including a sense of helplessness when confronting chronic, daily pain, and the unique stigma surrounding FM’s status as a legitimate medical condition (16, 17). Of the little research that has been done on this stigma, one study found that female patients reported being challenged regarding whether or not they were truly experiencing pain, as well as being accused of avoiding work due to their allegedly feigned illness (17). Thus, FM patients’ daily struggle with chronic pain, as well as the unique stigma, suspicion and lack of validation by large parts of the medical community may at least partly explain FM’s high comorbidity with depression, which in turn raises concern for suicide among FM patients. Despite this high comorbidity, however, research on FM and suicidality has been limited.

Suicidology research, irrespective of FM, classifies suicidality into a variety of categories. Such categories include non-suicidal self-injury, defined as deliberate attempts to harm oneself without an intention to die (18), suicidal ideation (SI), defined as thoughts of suicide in the absence of suicidal behavior (SB) (19), suicide attempts, and completed suicide (20). It must be noted that these categories do not necessarily lie on a spectrum of severity, but instead represent distinct and separate presentations of suicidality that differ categorically not only *via* cognitive, affective, or behavioral symptomatology, but also in associated risk factors. Thus, in the present review, we separate out these risk factors for SI and SB among FM patients. The intent to die is an important distinguishing factor between these facets of suicidality. It is what differentiates between passive and active SI, i.e., the passive desire to not be alive versus the active wish to die in the near future (21, 22). It also distinguishes between suicide attempts, intended to die, and self-harm, intended to communicate or relieve unbearable distress (23). While some research suggests that self-harm and suicide attempts differ categorically by intent to die (24), suicidology research more broadly has identified a variety of mechanisms by which suicidal ideation can shift to suicidal behavior apart from lethal intentions (e.g., emotion dysregulation, hopelessness) (25, 26). Other important factors include the frequency of the suicidal thought or behavior, as well as whether any SB resulted in injury (20).

Suicidology literature among chronic pain conditions focuses more generally on the distinction between suicidal thoughts and behaviors. Research supports a linkage between chronic pain conditions, such as migraine and non-migraine headaches, arthritis, rheumatism, and back problems with suicide ideation, attempts, and completed suicide (27–29). However, while research does exist on the relationship between chronic pain and suicide, studies specifically focusing on FM have been scarce, even though widespread chronic pain is the central feature of the disorder (4). Additionally, while extant research does indicate that there is an increased risk for suicidality in FM patients, the underlying factors contributing to suicidality among this population have yet to be understood. This paper aims to review the literature on FM and suicidality, in order to understand both its prevalence and correlates. To the best of our knowledge, this would be the first-ever comprehensive review of FM and suicidality to appear in the literature.

## Method

### Paper Search Strategy

The EBSCO Discovery Service, containing ScienceDirect, Medline, PsycArticles, and JSTOR, as well as Google Scholar, were used to identify relevant studies. The search terms used were “suicide”, “suicidality”, “suicidal”, or “suicid*” as coupled with “fibromyalgia”. Studies were included for the review if they explicitly measured either SI, attempted suicide, and/or completed suicide among FM patients. In this review, articles were included that studied patients suffering from FM in any country and within any age range. All studies included published data from a peer-reviewed journal. Notably, one study was included that was only available in abstract form (30). While the current review is based predominantly on peer-reviewed articles, this abstract was included as it contained relevant results obtained from a large sample size in the highly-neglected field of FM and suicidality.

There were several exclusion criteria for the present review. Case studies were excluded from the search. Studies that examined FM solely in its relation to non-suicidal self-injury (NSSI) were additionally excluded, as research has shown NSSI to be an independent (if related) phenomenon from suicide (31). Of note, one study which examined NSSI was included in this review (13), since it also examined suicide ideation and attempts. Studies and meta-analyses that did not specifically measure FM directly and distinctly were not included. This included, for example, studies that looked generally at chronic pain conditions without analyzing FM as a separate diagnostic condition, thereby not allowing for any FM-specific conclusions. Finally, studies that assessed the effectiveness of a medication on suicidality among FM patients were excluded as they did not add to the literature on the relationship itself between FM and suicidality.

**Figure 1:**
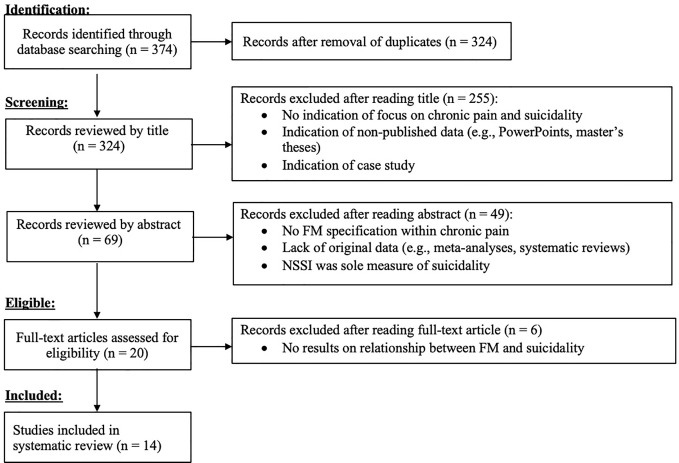
Flow chart detailing selection of included studies, according to Preferred Reporting Items for Systematic Reviews and Meta-Analyses (PRISMA) guidelines.

In terms of search methodology, one author initially identified 324 articles (excluding duplicates) which were screened for relevance to FM/suicidality. Any discrepancies identified at this stage were reviewed with the other author for discussion on inclusion of the study in the present review and discussed as based on the aforementioned inclusion and exclusion criteria. After excluding 49 articles following screening *via* abstract review, one author read the remaining full-text articles and discussed any studies that were not clearly defined by criteria with the other author until a conclusion was reached. See **Figure 1**, below, for a detailed figure describing the inclusion/exclusion process.

## Results

After applying the above mentioned inclusion/exclusion criteria, thirteen papers and one abstract (n = 14) were included in the present review that specifically examined the relationship between FM and suicidality. **Table 1** presents the studies included in the final review.

**Table 1:** Table compiling demographic and methodological details of reviewed studies.

Authors	Year of Publication	Sample Size *(N=)*	Age (standard deviation)Range	Diagnosis and Measure of FM	Measure of Suicide	Type of Suicide	Control Group	Study Design
Amir et al. (32)	2000	202	49 (8.4) FM 46 (13.1) Controls	Diagnosis based on ACR 1990 criteria^b^	Suicide Risk Scale	Suicide risk	Women with RA; women with LBP; healthy female controls	Cross-sectional
Calandre et al. (33)	2011	180	51 (8.5)	FIQR	Plutchik Suicide Risk Scale	Suicide attempt, suicide risk	No control	Cross-sectional
Calandre et al. (34)	2014	373	49 (8.6); 22–72	FIQ	Item 9 of the BDI	Passive & active SI	No control	Cross-sectional
Cheng et al.^a^ (30)	2009	5,982,904	Unknown	Diagnosis based on ICD-9^b^	Unknown	SB	Healthy controls matched by age and gender	Retrospective data analysis
Dreyer et al. (35)	2010	1361	19–70+^c^	Research team determined diagnosis of FM based on ACR 1990 criteria	Data pulled from Danish Mortality Register	Completed suicide	Patients with possible FM (diagnostic criteria not fully met)	Retrospective data analysis
Ilgen et al. (28)	2013	4,863,086	18–80+^c^	Diagnosis based on ICD-9, Clinical Modification^b^	Data pulled from NDI for date and cause of death and ICSD 10^th^ Edition for suicide mortality	Completed suicide	No control	Retrospective data analysis
Jimenez-Rodriguez et al. (36)	2014	126	55 (12.7) FM 51 (7.5) Controls	Diagnosis based on ACR 1990 criteria^b^	Item 9 of the BDI; Plutchik Suicide Risk Scale	Passive and active SI; suicide risk	Patients with LBP; healthy controls	Cross-sectional
Lafuente-Castro et al. (37)	2018	102	52 (8.2)	Diagnosis based on ACR 1990 criteria	Item 9 of PHQ-9; Plutchik Suicide Risk Scale	SI; suicide risk	Healthy controls	Cross-sectional
Lan et al. (13)	2016	285,449	<35 – >65^c^	Diagnosis based on ICD-9^b^	Assessed *via* ICD-9-Clinical Modification codes	Suicide attempt; completed suicide; NSSI	Matched reference controls based on age, sex, and index year	Retrospective data analysis
Liu et al. (38)	2015	1,318	44 (12.6) Migraine/comorbid FM 15–98^c^	Questionnaires based off of ACR 2010 criteria	Self-reports of lifetime suicide ideation and attempts	SI; suicide attempt	No control	Cross-sectional
McKernan et al. (39)	2018	8,879	57 (14.1) SI cases	Diagnosis *via* electronic algorithm (PheKB)	Data pulled from PheKB based on ICD, 9^th^ edition	SI; suicide attempt	Healthy controls	Case-control
Ratcliffe et al. (29)	2008	36,984	15–65+^c^	Clinical interview with diagnosis from health professional	Personal interviews	SI; suicide attempt	No control	Cross-sectional
Triñanes et al. (40)	2014	117	49 (9.3); 22–80	Diagnosis based on ACR 1990 criteria; FIQR	Item 9 of the BDI	SI	No control	Cross-sectional
Wolfe et al. (41)	2011	8,186	51 (12.4)	Diagnosis from rheumatologist based on ACR 2010 criteria; FMness Scale	Data pulled from US National Death Index; NDB	Completed suicide	Patients with osteoarthritis	Retrospective data analysis

ACR, American College of Rheumatology; RA, rheumatoid arthritis; LBP, lower back pain; FIQ, FM Impact Questionnaire (42); FIQR, FM Impact Questionnaire – Revised (43); BDI, Beck Depression Inventory (44); ICD-9, International Classification of Diseases, 9^th^ Revision; NDI, National Death Index; ICSD, International Statistical Classification of Diseases; NDB, National Data Bank for Rheumatic Diseases; PheKB, Phenotype KnowledgeBase; PHQ-9, Patient Health Questionnaire – 9.

a Solely the abstract is published, thus only information contained within it is included within this table and the entirety of the review.

b The study did not specify how diagnosis was made. The only information specified was the diagnostic criteria.

c No mean age of the sample was reported. Instead, we reported the range.

### General Study Characteristics: Variables Assessed, Demographics, and Methodology

As can be seen in **Table 1**, a range of suicidality variables was assessed. Two studies solely assessed SI (34, 40), three solely investigated completed suicide (28, 32, 41), and one study examined solely suicide risk (32), which was defined as thoughts of suicide, depression, and hopelessness, among other variables (45). The remaining portion of the reviewed studies examined a multitude of suicidal thoughts and behaviors.

In terms of demographics, sample sizes and study designs, studies were heterogeneous. Sample sizes ranged from mid-sized (N = 117) to extremely large (N = 5,982,904) (25, 34). In terms of gender representation, the majority of the studies’ samples were heavily weighted towards the female gender. Two studies, in fact, utilized samples that were fully comprised of females (32, 35).

Age range generally remained similar throughout most studies, representing an adult population between the ages of 18 to over 80 years old, except for one study which included adolescents in addition to adults in the study sample (29).

In terms of methodology, study designs were primarily cross-sectional or retrospective cohort. Notably, no longitudinal studies could be found. Most studies included patients solely with FM; interestingly, one study examined patients with both diagnosed and “possible” FM, in which FM diagnostic criteria were not fully met (41), and another study examined patients with migraine and comorbid FM (38). Studies utilized a variety of control groups, varying from matched reference controls (13), to patients with low-back pain (LBP) (36) and osteoarthritis (OA) (30), to healthy controls (32, 35).

The majority of studies utilized self-report measures to garner information about symptoms of FM, physical pain, and suicidality. FM was typically assessed either through a physician’s diagnosis based on ACR or ICD-9 criteria or through use of self-report measures. Five out of the 13 studies utilized self-report questionnaires to assess suicidality. Three studies utilized national databases such as the NHIRD, the National Death Index (NDI), and the Danish Mortality Register to assess for completed suicide. In terms of measuring physical pain, the majority of studies again utilized self-report measures such as the Visual Analogue Scale or the Widespread Pain Index. Only one study utilized a more objective measure of pain, i.e., pain threshold and tolerance were measured by applying a pressure algometer on reported tender points and measuring the minimum and maximum forces that yielded pain to the participant (40).

### Prevalence of SI and SB Among FM Patients

Results from the included studies strongly indicate that SI was prevalent among individuals suffering from FM. This finding holds for studies that specifically examined samples with no healthy controls. One study that solely examined patients with FM found that 48% of FM participants exhibited SI, 39.7% of which was passive and 8.3% of which was active (34). In a cross-sectional study that utilized a sample of migraine and comorbid FM patients, it was found that 58.3% of patients suffering from both conditions exhibited SI. It was additionally found in this study that patients with both migraine and comorbid FM exhibited significantly more SI than patients with only migraine, and that comorbid FM was an independent predictor of SI among patients with migraines (38). Results from a study examining solely women with FM, it was found that 26.5% of women with FM had SI, 6% of which was active (40).

Prevalence rates were similarly high among studies with which samples of both FM and healthy controls were utilized. Among a study examining both patients with FM and healthy controls, results indicated that 28.3% of FM patients exhibited SI, as compared to healthy controls who exhibited no SI. Additionally, the odds ratios for SI among FM patients were significantly higher than that of healthy controls (37). Interestingly, among a sample of either FM patients, lower back pain patients, or healthy controls, it was found that FM patients had significantly more passive and active SI than patients with either lower back pain or no pain, after adjusting for age and gender (36). In contrast, one case-control study of almost 15,000 suicide attempters at a major US hospital as compared to general patients at the hospital found that only 1.1% of the FM sample exhibited SI (39).

>Results from the included studies also strongly indicated that SB was prevalent among individuals suffering from FM. First, we examine studies that did not utilize healthy controls in their sample. In a cross-sectional study examining 1,318 patients with migraine and comorbid FM, 17.6% of patients with both chronic pain conditions reported attempted suicide, a rate significantly higher compared to that found among patients with only migraine. Additionally, it was found that comorbid FM was independently associated with suicide attempts in patients with migraine, even after controlling for demographics, headache characteristics, and effects related to psychopathology and daily functioning (38). Among a sample of Spanish patients with FM, 16.7% of patients reported previous suicide attempts. Among these patients, 66.7% reported one suicide attempt, 16.7% reported two suicide attempts, and 16.7% reported three suicide attempts, with the preferred method of attempt being drug poisoning (70%). Additionally, among FM patients who attempted suicide, 38.9% required emergency hospitalization. Interestingly, FM severity scores were significantly higher among suicide attempters as compared to non-attempters. However, pain scores did not differ between the groups (33). Finally, it was found that the standardized odds ratio for completed suicide among FM patients and osteoarthritis patients as compared to the US general population was 3.31 (41).

Results also indicate that SB was prevalent among studies that utilized healthy controls. In a mortality study of a cohort of patients with both confirmed and “possible” FM (i.e., FM diagnostic criteria were not fully met), there was a standardized mortality ratio of 10.5 as compared to the general population. Additionally, it was found that patients with diagnosed FM had a significantly higher risk of completing suicide than patients with “possible” FM (35). In a study examining patients with a variety of chronic pain conditions (including FM) as well as healthy controls, rates of SB were not reported among FM patients specifically, but FM was found to be associated with a higher risk of completed suicide among a sample of veterans with general pain conditions. However, this association became non-significant after controlling for age, gender, medical comorbidities, and psychiatric comorbidities (28). Interestingly, McKernan and colleagues’ (39) large scale study examining suicide attempters as compared to the general patient population at a major US hospital indicated that only 0.4% of FM patients reported suicide attempts. Similarly, another study examining almost 200,000 patients with a variety of chronic pain conditions, as well as healthy controls, found that only 31 FM patients per 100,000 person-years [i.e., the number of participants multiplied by the length of time the participants were followed for (46)] exhibited SB (32).

In a study examining suicide risk, defined as a combination of the presence of SI, past suicide attempts, and related psychological factors and symptoms, it was found that 81.8% of FM patients were at risk for suicidal ideation and behavior, which was significantly higher than that of patients with low-back pain and healthy controls (32). Amir and colleagues (32) also examined suicide risk, as described above, and found that there was a descriptive difference in suicide risk between FM (M = 44.5, SD = 8.4) and healthy controls (M = 46.8, SD = 3.3), however, this difference did not reach statistical significance, p = .072. Lafuente-Castro and colleagues (37) also found that odds ratios for suicide risk among FM patients were significantly higher than that of healthy controls.

Next, we will present findings regarding factors associated with elevated levels of SI and behavior among individuals with FM. Because the literature commonly categorizes SI and SB as separate phenomena along the continuum of suicidality, results of will be described separately. It should be stressed, that since none of the FM suicide studies were based on a longitudinal study design, no causal relationships could be established between a certain factor and SI or SB.

### Factors Associated With SI Among FM Patients

#### Psychiatric Comorbidity

Several studies indicate that psychiatric comorbidities play an important role in SI among those diagnosed with FM. Interestingly, after controlling for any mood, anxiety, substance dependence disorder, or any other mental disorder, one study found that FM and SI were no longer associated (29), thus supporting the important role of comorbid psychiatric conditions in suicidality among FM patients.

Depression in particular was commonly found to be associated with SI among FM patients (32, 34, 38, 40). In one study, depression was found to be an independent predictor of SI in patients with both migraine and comorbid FM (38). In an attempt to further explain the relationship between FM, SI, and depression, Triñanes and colleagues (40) examined the unique roles of the three depressive symptom clusters of the Beck Depression Inventory (BDI; 44), indicating that self-blame was the only independent predictor of SI in FM patients. This study additionally found that FM patients with SI were significantly more likely to be depressed than FM patients without SI (40). An additional study corroborated these results, finding that FM patients with SI scored significantly higher on the BDI than FM patients without SI (34). Also, in the realm of mood disorders, a study by McKernan and colleagues (39) found an elevated risk of SI among FM patients diagnoses with Bipolar Disorder-Not Otherwise Specified compared to those without this comorbid disorder (39).

Anxiety was also assessed with regards to SI, with studies showing that FM patients with SI were significantly more likely to suffer from anxiety compared to FM patients without SI (34, 40). Additionally, it was found that FM patients with SI exhibited significantly more anxiety than FM patients without SI (34). In one study, anxiety was also independently associated with SI specifically among patients with migraine and comorbid FM (38).

#### Sleep Difficulties

Liu and colleagues (38) found that poor sleep quality was independently associated with SI in patients with migraine and comorbid FM. Other studies have shown that FM patients with SI reported significantly higher daytime dysfunction due to sleepiness (40), fatigue (39), and poor sleep quality (34) when compared to FM patients without SI. Calandre and colleagues (34) similarly found significantly elevated levels of fatigue and poor sleep quality when comparing FM patients without SI, with passive SI, and with active SI, with the highest levels of reported fatigue and poor sleep quality among FM patients with active SI and the lowest levels among FM patients with no SI.

#### Chronic Pain

Although pain is the core feature of FM, evidence regarding its association with SI is surprisingly scarce and highly inconsistent. One study found that FM patients with SI reported more pain than FM patients without SI, but this association did not reach significance at the.01 level (34). Corroborating this finding, another study found no significant differences in bodily pain, pain threshold, pain tolerance, or interference of pain with work among FM patients with and without SI (40). Finally, a study by Liu and colleagues (38) showed that headache frequency was independently associated with SI in patients with migraine and comorbid FM (35).

#### Specific FM Somatic Symptoms

McKernan and colleagues (39) found that dizziness and weakness were both associated with increased SI among FM patients. Odds ratios were 1.25 and 1.17, respectively, when compared to the general population of the Vanderbilt University Medical Center.

#### Impairment of Functioning

As FM often entails impaired daily functioning, some studies have assessed the role of functional impairment in SI severity. Studies have found that increased functional impairment in day-to-day life often differentiates between FM with and without SI. One study found that FM patients with SI reported significantly more impairment of functioning due to sleepiness compared to FM patients without SI (40). It was additionally found that FM patients with SI missed significantly more work (i.e., occupational functioning) than FM patients without SI (34).

#### Inpatient Hospitalization

It was found that the number of inpatient hospitalizations within the past year was positively associated with SI levels among FM patients (39). It must be noted that it was not made clear whether the hospitalizations were psychiatric in nature or referred more generally to any type of inpatient hospitalization. Interestingly, frequent follow-up visits at outpatient clinics, as well as increased outpatient prescriptions—both psychiatric and medical in nature, were associated with less SI.

### Factors Associated With SB Among FM Patients

We will now present findings related to factors associated with SB. In general, these findings are even more scarce compared to those related to SI.

#### Psychiatric Comorbidity

Depression was found to be independently associated with suicide attempts in patients with migraines and comorbid FM (38). Recurrent depression with psychosis, interestingly, was also found to be associated with suicide attempts among FM patients (39). Similarly, other studies found that FM patients who attempted suicide were significantly more likely to have depression and anxiety when compared to FM patients who did not attempt suicide (33). Notably, while FM was associated with suicide attempts, after controlling for mood disorders, anxiety disorders, substance dependence disorders, and any other mental disorder, this association no longer existed (29). Finally, another study found that after controlling for comorbid psychiatric conditions, as well as age and sex, the association between FM and completed suicide no longer existed (28). Interestingly, a Danish study showed contrasting results; Dreyer and colleagues (35) found that no participant from the FM sample was diagnosed with any other psychiatric condition, suggesting that comorbid psychiatric conditions could not explain the relationship between FM and suicidality due to the lack of comorbidity to begin with among the sample.

#### Gender

There are mixed findings with regard to the role gender may play in the association between FM and SB. While one study found that being female was associated with a higher risk of completed suicide among FM patients, another study found no gender difference in the risk for completed suicide, suicide attempts, and NSSI among patients with FM, after controlling for age, occupation, income, comorbidities, and non-steroid anti-inflammatory drugs (13, 35). However, Lan and colleagues (13) did find that being female was a significant factor associated with attempted suicide, completed suicide, or NSSI among patients younger than 35 (hazard ratio = 1.54) as well as patients between the ages of 35–65 (hazard ratio = 1.37).

#### Employment Status and Income

In terms of occupation, significantly more office workers with FM attempted suicide, completed suicide, and were involved in NSSI (11). Additionally, significantly more individuals with FM who had a monthly income of over 25,000 Taiwanese dollars, roughly equivalent to only $829.28 a month, which is below minimum wage in Taiwan (47 ^1^, 48 ^2^), were at risk for attempted suicide, completed suicide, and NSSI (13), suggesting a potential linkage between low socioeconomic status and suicide in FM. Finally, FM patients were found to attempt suicide more if they were unemployed or on sick leave as compared to non-attempters (33). However, after controlling for other demographics such as gender, education, marital status, and age, FM was still associated with suicide attempts, implying that demographics may not explain much of the variance underlying the relationship between FM and suicidality (29).

#### Sleep Difficulties

Lan and colleagues (13) found that the risk of NSSI, attempted suicide, and completed suicide was significantly higher in patients with a sleep disorder as compared to patients without a sleep disorder. It was also found that FM patients who attempted suicide were significantly more likely to experience sleep problems than FM patients who did not attempt suicide (33).

#### Medical Comorbidity

It was found that risk of attempted suicide, completed suicide, or NSSI in patients with FM without comorbidity was significantly higher than non-FM patients also without comorbidity (13). Headache frequency, specifically, was found to be independently associated with suicide attempts in patients with migraines and comorbid FM (38). Obesity was additionally found to be associated with suicide attempts among FM patients (39).

#### Drug Dependence

Only one study examined the role of substance abuse in suicidality in FM. Cocaine dependence was determined to be associated with suicide attempts among a sample of FM patients (39). This finding was determined *via* a novel machine learning algorithm designed to detect risk of suicide attempts (49), that was subsequently applied to FM patients (39).

#### Inpatient Hospitalization

McKernan and colleagues (39) explored whether or not inpatient hospitalization was indicative of help-seeking behaviors that could protect against SB, or alternatively of severe suicidality (i.e., the patient was at safety risk and thus at-risk for suicide attempts). Results showed that the number of inpatient hospitalizations in the past year was an associated with suicide attempts among FM patients. Interestingly, increased outpatient clinic follow-ups and outpatient prescriptions served as protective factors against suicide attempts among FM.

## Discussion

FM is a unique condition, accompanied by a wide variety of daily difficulties. From symptoms of chronic pain to cognitive impairment and social stigma, individuals with FM are in a constant state of physical and psychological suffering (2, 7, 17). It is due to these adversities that a greater understanding of suicide risk in FM is needed.

The present literature review had three aims. The first aim was to illustrate the prevalence of suicide-related outcomes among FM patients. Second, the review sought to highlight factors associated with SI and behavior that have been identified in the literature. Finally, this review aimed to identify gaps in the current state of the literature regarding SI and behavior among this population.

### Prevalence of SI and Behavior in FM

Prevalence rates for both SI and behavior varied widely across reviewed studies. Prevalence rates ranged from 1.1% (39) to 58.3% (38) for SI among FM patients. This wide range indicates that more work needs to be done to better understand the pervasiveness of suicidality in this condition. Interestingly, McKernan and colleagues’ (39) finding of a 1.1% prevalence rate of suicidal ideation in FM patients may be due to assessing SI *via* a clinician’s diagnosis, as opposed to self-reports, on which other reviewed studies relied (34, 36, 38). While it may be speculated that the similarly low prevalence found by Cheng and colleagues (30) might be due to similar reasons, conclusions cannot be made due to the lack of methodological information presented in the published abstract. Previous research suggests that patients disclose more SI *via* self-report as compared to face-to-face clinician screenings (50), which may explain McKernan and colleagues’ (39) lower outcome prevalence. If the results of McKernan and colleagues (39) are excluded, then the range becomes less wide, ranging from 26.5% (40) to 58.3% (38).

In terms of SB, prevalence rates once again widely varied, ranging from 0.4% (39) to 17.6% (36). This seemingly large range is likely explained by the fact that McKernan and colleagues (39) solely studied suicide attempts 30 days after a patient’s last hospital visit, whereas Calandre and colleagues (33), as well as Liu and colleagues (38), assessed a lifetime history of suicide attempts. While McKernan and colleagues (39) found a very low prevalence of 0.4% as compared to that of Calandre and colleagues (33) and Liu and colleagues (38), at 16.7 and 17.6%, respectively, this difference may be accounted for by the fact that lifetime history of suicide attempts would likely be higher than that of suicide attempts solely 30 days after a patient’s last hospital visit. Dreyer and colleagues (35) alternatively only examined prevalence rates of completed suicide, although their standardized mortality ratio of 10.5 suggested a 10-fold increase in the risk of suicide among FM patients as compared to the general population. Additionally, an odds ratio of 3.31 was found for completed suicide among FM as compared to the general United States population (41). In addition, only 31 FM patients per 100,000 person-years exhibited SB (30). These differences in prevalence rates point to the need for more standardization of assessments across both SI and behavior.

Overall, while prevalence rates varied between studies, it seems largely agreed upon that prevalence rates for suicide-related outcomes among FM patients are high as compared to the general population, in which overall prevalence of suicidal thoughts is 4.3% and overall prevalence of SBs (i.e., suicide attempts) is 0.6% (51). Thus, the FM population deserves particular attention. However, future studies are encouraged to employ more standardized ways of assessing suicidality, in order to achieve more robust and stable results. A more accurate evaluation of the true scope of suicidality in FM may also contribute to its socio-medical validation as a legitimate condition, which causes severe distress to those diagnosed.

### Factors Associated With SI and SB Among FM Patients

Next, we will discuss factors identified in the literature as associated with SB and/or ideation in individuals diagnosed with FM. In an attempt to better integrate the reviewed findings, we have identified two groups of factors: those that were found to be associated with *both* SI and SB, and those that were associated with only one, but not the other. We will proceed by discussing these groups of factors separately.

### Factors Associated With Both SI and SB in FM

In this section, we will discuss factors found to be associated with both SI and behavior. The fact that they are related to both aspects of suicidality may indicate that they play a particularly important role among FM patients, and thus call for special consideration and attention by mental health professionals.

Unsurprisingly, psychiatric comorbidity, namely depression and anxiety, was commonly found to be associated with both SI and behavior among FM patients. As noted, previous research indicates that FM is highly comorbid with mental disorders, such as depression and anxiety (52), bipolar disorder (53), and posttraumatic stress disorder (54). In fact, some may argue that FM is, by definition, a complex, heterogenous condition, where physical and psychiatric symptoms never appear in isolation. Thus, speaking of “comorbidity” may be somewhat misleading in the case of FM, as comorbidity is the rule rather than the exception. In line with that notion, it seems that it is not merely the strictly rheumatic symptoms of FM that are associated with suicidality, but rather its accompanying psychiatric symptoms. The negative mood, as well as increased anxiety and stress, often experienced by this population may in turn yield increased SI and behavior.

Because psychiatric comorbidity is so frequently highlighted in FM studies, therapies that target depression, anxiety, and other comorbid psychiatric conditions can potentially reduce SI and behavior. For example, results of one RCT reported that a treatment program consisting of physical activity, psychoeducation, and self-management techniques effectively reduced symptoms of depression and anxiety among individuals with FM (55). In addition, educating FM patients about the prevalence of psychiatric comorbidity may normalize a patient’s experience and potentially function to help patients’ pains, anxieties, and other concerns feel validated (56). Psychopharmacology may be relevant for FM and accompanying disorders as a method of alleviating suicidality (57). However, research is inconclusive regarding whether or not psychopharmacological medications can actually prevent suicide (58, 59). Finally, we should note again that since all reviewed papers were cross-sectional or relied on retrospective reports, we cannot exclude the possibility that SI and SB have strongly *contributed* to elevated levels of depression and anxiety. Thus, the directionality of results cannot be determined, as will also be noted regarding other findings in this review.

Because psychiatric comorbidity was indicated to be commonly associated with both SI and SB, it is unsurprising that sleep difficulties also related to SI and SB. Generally speaking, sleep is a common mechanism underlying psychopathology (60). In the case of FM, impaired sleep may serve as an important axis connecting pain, fatigue and psychiatric distress. Because difficulties with sleep have been heavily associated with psychiatric distress, as well as suicidality (61), and since chronic fatigue is a central feature of FM (4), sleep difficulty may contribute to both SI and SB. Disturbed sleep was often found to be associated with increased levels of depression and agitation, as well as decreased health-related quality of life (62). Hopelessness in particular has been found to mediate the association between insomnia and SI (63) and thus may act as a more specific risk factor. Thus, sleep therapy should be considered as a form of treatment for FM patients. Cognitive behavioral therapy for insomnia has been shown to be effective at treating sleep difficulties among FM patients (64), and could potentially be useful to reduce suicidality in FM. Once again, it is also quite possible that impaired sleep is a consequence of suicidality and overall declining mental health, and this directionality should be taken into account as well.

In discussing SI/SB and FM in association with psychiatric comorbidity, fatigue, and pain, finding that inpatient hospitalization is a risk factor for both forms of suicidality is unsurprising. This finding may be attributed to the notion that hospitalization signifies increased pain and FM symptom severity that call for immediate attention. Among a general FM population, one service utilization study found that a single FM patient would be hospitalized approximately one time every three years. It was additionally found that almost 50% of hospitalizations among FM patients could be attributed to FM-related symptoms, with patients being admitted primarily due to musculoskeletal, neurological, gastrointestinal, cardiovascular, or depressive symptoms (65). Of these primary reasons for hospitalization, all but gastrointestinal issues are associated with increased risk of suicidality (66–69). Such a high frequency of hospitalizations for FM-related symptoms associated with suicidality is particularly concerning and illustrates why more attention needs to be paid to this high-risk population. Additionally, past research indicates significantly increased inpatient psychiatric hospitalizations visits prior to suicide-related outcomes, attributed primarily to a diagnosis of an affective disorder, as well as a history of psychiatric illness (70). Thus, it is not surprising that inpatient hospitalizations were cited as associated with suicidality in this population.

### Factors Associated With Only SI or SB in FM

Interestingly, many of the core clinical symptoms of FM were found to be more strongly associated with SI than with SB. Chronic pain and fatigue, which are both core symptoms of FM (2, 4), were associated with increased SI among FM patients (34). Similarly, dizziness and weakness, two neurological symptoms associated with FM (71), were also associated with increased SI (39). Living with chronic pain, weakness, dizziness, and fatigue is highly frustrating, and may deplete one’s psychological resources. Thus, it is not surprising that these symptoms are associated with increased SI. The literature, in fact, indicates associations between chronic pain and fatigue with hopelessness (72, 73), which, in turn, may lead to suicidal thoughts (19). The fact that chronic pain was not found to be associated with SB is somewhat puzzling, especially given previous studies (not on FM), showing different results (e.g., 74). Presumably, these results may be attributed to the scarcity of suicide research in FM, as well as to the different methods of measuring pain across studies. However, the results stemming from this review may also indicate that it is not the core symptoms of FM per-se that drive one to suicide, but rather more “downstream” psychopathological effects of these symptoms, most notably major depression.

Unsurprisingly, drug dependence was also found to be common among individuals with FM (75), especially when including over-the-counter pain medications, which are commonly used by patients (76), as well as marijuana, which was found to be used among 13% of a sample of 457 FM patients (77). Interestingly, given that drug abuse has been widely cited as a risk factor for SI and behavior (78, 79), the result that only cocaine abuse was associated with suicide attempts among FM patients (39) was surprising. However, viewing this finding in its own right, it is not surprising that cocaine abuse was associated with SB given previous research citing it as a risk factor for suicidality regardless of an FM diagnosis (80). Santis and colleagues (81) similarly found that, even within cocaine users, there exists a spectrum of cocaine abuse in which some are more likely than others to self-harm and attempt suicide, namely individuals who use cocaine base paste as compared to cocaine hydrochloride. Finally, due to the cross-sectional nature of studies presented here, it is highly possible that SB, and the emotional pain associated with it, in fact lead to increased drug dependence, as form of self-medication (77). Nonetheless, cocaine and other drug abuse may be extremely dangerous among FM patients, given its high mortality rate regardless of an FM diagnosis (82). Thus, the treating physician or mental health professional is encouraged to be particularly attuned to issues relating to drug abuse among FM patients. Additionally, these clinicians should put an emphasis, if needed, to combine strategies to manage addiction in addition to FM.

In addition to drug abuse and psychosomatic symptoms in FM, medical conditions such as stroke (83) and chronic abdominal pain (84) also were shown to be associated with increased risk of SB, regardless of an FM diagnosis. It must be noted that gastrointestinal symptoms, previously cited as not being associated with suicidality (66–69), are a specific subset of abdominal pain. This suggests that these results are not discrepant; rather, they suggest that whereas gastrointestinal symptoms specifically may not be indicative of suicidality, other chronic abdominal pain symptoms may be (84). It is of no surprise that a medical condition that is comorbid with FM was associated with SB among diagnosed individuals. This may be seen as an additive effect, i.e., the physical and emotional suffering characterizing FM is joined by additional distress caused by any of these accompanying conditions. The body is at the core of FM patients’ distress, and thus any additional physical difficulty might render the pain unbearable and increase one’s wish to end life.

Results of the current review also demonstrate that impairment of functioning was associated with SI among FM patients (34, 40). It is important to note that due to the cross-sectional nature of the reviewed studies, there is no clear way of establishing causality in the association between impairment of functioning and SI in FM. Nonetheless, FM has severe effects on daily functioning (85). The pain and fatigue very often undermine one’s ability and motivation to conduct even the simplest of daily tasks, including driving, visiting family and friends, and shopping for groceries. Thus, daily life may become constricted, and this constriction in turn may yield highly negative thoughts and beliefs about life. Psychological interventions should therefore focus on maintaining daily functioning when treating FM patients, as a means to improve mood, motivation, and vitality.

Given the association found between day-to-day functional impairment and suicidality in FM, an association between employment status and suicidality in this population makes sense; unemployment may be seen as an extreme point on the continuum of daily dysfunction (see above). It was found that both difficulty with employment (33) and an income below minimum wage (13, 47) were associated with SB among FM patients. These findings are in line with previous research, which shows that both unemployment (86) and being poverty-stricken (87) have been linked to SB, regardless of an FM diagnosis. One’s work often provides an important anchor for functioning, self-worth and sense of potency, and thus being unemployed may significantly increase the risk of SB. This is true especially among individuals suffering from conditions such as FM, where the symptoms themselves may have significantly undermined one’s ability to work, yielding self-blame, hopelessness and despair. Thus, as noted by Henriksson and colleagues (88), there is a pressing need to identify factors which may contribute to FM patients’ ability to remain in the job market despite potential difficulties, as well as to find ways to adjust work conditions for those with partial work ability to meet their existing physical and psychological abilities. Once again, due to the cross-sectional designs of all reviewed studies, one cannot rule out the possibility that SB affects employment status as well, with those attempting suicide being unable to work, keep a job, or earn a sufficient living.

In line with previously discussed psychosocial risk factors, gender was another factor found to be associated with suicidality. Interestingly, one study showed that being female was associated with a higher risk of completed suicide (34). This stands in contrast to much of the literature, where men were often shown to face a higher risk for completed suicide (89). However, the finding from Lan and colleagues (13) that there was no gender difference in completed suicide risk can potentially support this hypothesis, as no gender difference still shows an increase in the amount of females who complete suicide when compared to national averages, where males complete suicide more than females (89). These findings may be linked to previous studies showing that women suffered from more severe symptoms of FM as compared to men (90), which may, in turn, lead to increased levels of emotional distress and completed suicide. In addition, the finding may be attributed to issues of illness identity and perception. FM has been known to be a highly “feminine” disorder, with a clearly imbalanced female-to-male morbidity ratio. As noted by Briones-Vozmediano (91), there may be a social bias against women as far as FM diagnosis goes. Applying gender theory, the authors show how gender stereotypes may influence the social construction of FM. Thus, it may be that SB is in fact not higher among women with FM, but rather that they often receive the FM diagnosis much more quickly than men, creating a skewed perspective regarding suicidality. Another possibility is that once a woman is already diagnosed with FM, she is often met with increased suspicion and skepticism, which, in turn, may increase her emotional distress (17). Interestingly, there were mixed findings related to the role of age with regard to gender and SB among FM patients. Thus, one study found no gender differences when controlling for age (34), whereas another found significant gender differences in the risk of SBs for FM patients 65 years or younger (13). This finding could potentially be explained by an increase in comorbidities and duration of FM symptoms as people age (92). Generally speaking, these finding highlight the need for more comprehensive and systematic studies of mixed-gender FM samples, in an attempt to disentangle the issue of gender differences in FM suicidality. In addition, mental health professionals are encouraged to pay attention to gender differences when treating those with FM, as well as to the specific needs and perceptions of women diagnosed with this condition.

### Summary and Future Steps

FM is not well understood. While the disorder can be traced to dysfunction of the central nervous system, its underlying mechanisms are not clear, and it has widespread comorbidities in both mental disorders and medical conditions (4, 13). Additionally, definitions of FM are constantly being revised, highlighting the widespread difficulty in defining and diagnosing this disorder.

As a result of this lack of clarity, patients often experience stigma (16, 17), which strongly affects their emotional well-being. In addition, FM patients are up to 60% more likely than the general population to experience depression (15) and are up to 58.3 and 17.6% more likely to experience SI and behavior, respectively (38). In addition to the higher risk of suicidality, FM patients are at higher risks of psychiatric and medical comorbidities, such as drug abuse, that are independently associated with increased risk of suicidality (52–54). Thus, it is vital to better understand suicide among this high-risk population in order to prevent future deaths.

First and foremost, this review indicates the surprising scarcity of research in the area of FM and suicide. This scarcity stands in sharp contrast to what is known about FM and its accompanying distress. Much more research is called for in order to understand the scope of SI and SB among individuals with FM, as well as the factors contributing to them. In our review, we have decided to separately discuss shared and non-shared factors for SI and SB. Looking at factors contributing to both ideation and behavior, psychiatric comorbidity seems to be a particularly important factor. Thus, comprehensive psychiatric assessments of FM patients are needed in order to tailor interventions targeting depression, anxiety, stress and substance abuse, with the goal of preventing future suicide attempts. Sleep also seems to be a major risk factor, a finding which calls for improved and novel sleep interventions, alleviating the fatigue of those diagnosed with FM. Importantly, our review indicated that FM interventions should ideally be interdisciplinary and holistic in nature, comprising of a team of physicians, mental health clinicians, and allied health professionals to ensure effective and sustainable suicide prevention. As the risk factors are highly heterogenous, so must be the treatment.

Methodologically, our review reveals much room for improvement in future studies. Future research should focus both on replicating and expanding on findings from the reviewed studies, as well as addressing the studies’ major limitations. Importantly, no study on suicidality within FM has assessed suicide risk factors longitudinally, thus excluding the possibility of drawing important causal conclusions. For example, only one study found that suicidality may exacerbate FM symptoms (33). More studies are needed to better understand this phenomenon, as it points to the possibility of a vicious cycle connecting FM and suicidality, which is highly understudied. In addition, studies applying Ecological Momentary Assessments (93) may increase external validity and thus strengthen results of FM suicide studies. Future studies should explicate exactly how indices of suicidality were assessed (e.g., self-report, clinician-rated). The assessment of pain and its role in FM-related suicide also needs to be improved. Future studies should attend to novel methods of studying chronic pain more objectively (e.g., 94), as rather than rely solely or mainly on subjective self-report measures (4). Finally, future studies are encouraged to include biological and physiological measures of FM-related suicidality, as these measures were not used thus far in order to understand suicide risk among this population. In general, while the field of suicide research has massively evolved in recent decades, only few of its available research tools and methods have so far been applied to FM populations.

In terms of content and assessed variables, future studies are encouraged to take a much more in-depth approach when examining suicide-related psychological processes. Variables such as stigma, illness perception and identity, loneliness, and hopelessness may play an important role, and have hardly been studied. We argue that saying “depression” or “anxiety” alone may not be sufficient in order to inform mental health practitioners as to specific therapeutic targets among FM patients in risk of suicide. Finally, and in line with the latter point, we would encourage more qualitative studies of suicide in FM, in order to gain a first-person perspective of emotional pain and suicide proneness among this unique population.

## Author Contributions

DH conceived of the topic of review and supervised DL’s work throughout the process. DL conducted the literature search, identified the articles of interest, and summarized relevant findings. DL wrote the first and edited drafts of the manuscript under the supervision of DH. DH reviewed and edited all versions of the manuscript. All authors contributed to the article and approved the submitted version.

## Conflict of Interest

The authors declare that the research was conducted in the absence of any commercial or financial relationships that could be construed as a potential conflict of interest.

## Footnotes

1
https://www.bloomberg.com/quote/USDTWD:CUR

2
https://www.taiwannews.com.tw/en/news/3641344
